# Association of leptin G2548A and leptin receptor Q223R polymorphisms and their serum levels with infertility and recurrent pregnancy loss in Iranian women with polycystic ovary syndrome

**DOI:** 10.1371/journal.pone.0255920

**Published:** 2021-08-18

**Authors:** Fatemeh Bagheri Kargasheh, Soheila Ansaripour, Nasrin Borumandnia, Nariman Moradi, Zahra Zandieh, Monireh Maleki, Sara Mokhtar, Atousa Karimi, Farnaz Fatemi, Asma Kheirollahi, Akram Vatannejad

**Affiliations:** 1 Department of Biology, Tehran Medical Branch, Islamic Azad University, Tehran, Iran; 2 Reproductive Biotechnology Research Center, Avicenna Research Institute, ACECR, Tehran, Iran; 3 Urology and Nephrology Research Centre, Shahid Beheshti University of Medical Sciences, Tehran, Iran; 4 Cellular and Molecular Research Center, Research Institute for Health Development, Kurdistan University of Medical Sciences, Sanandaj, Iran; 5 Shahid Akbar Abadi Clinical Research Development Unit (ShACRDU), Iran University of Medical Sciences, Tehran, Iran; 6 Avicenna Infertility Clinic, Avicenna Research Institute, ACECR, Tehran, Iran; 7 Department of Comparative Biosciences, Faculty of Veterinary Medicine, University of Tehran, Tehran, Iran; CHA University, REPUBLIC OF KOREA

## Abstract

**Background:**

Adipokine leptin plays a crucial role in metabolic and reproductive functions. Leptin receptor has a soluble form that binds to leptin, thus modulating its level in the circulation. It has been indicated that the levels of leptin and leptin receptor and also *LEP* rs7799039 and *LEPR* rs1137101 polymorphisms are associated with metabolic disorders. In the present study, we assessed the levels of leptin and soluble leptin receptor (sOB-R), and also the frequency of rs7799039 and rs1137101 polymorphisms in healthy fertile women and patients with polycystic ovary syndrome (PCOS), inclusive of PCOS-infertile and PCOS-recurrent pregnancy loss (RPL) subjects.

**Methods:**

A total of 324 PCOS patients- including 199 infertile cases and 125 patients with a history of RPL- and 144 healthy controls were enrolled in this study. Biochemical parameters and plasma leptin and sOB-R levels were measured by ELISA and the genotypes of rs7799039 and rs1137101 polymorphisms were determined using PCR- RFLP.

**Results:**

Plasma leptin and sOB-R levels were significantly higher and lower in PCOS, PCOS-infertile and PCOS RPL groups, respectively. The GG genotype frequencies of rs7799039 and rs1137101 polymorphisms were significantly different between PCOS-infertile women and non-PCOS subjects (P = 0.043, OR = 0.47, 95% CI = 0.22–0.97, and P = 0.01, OR = 0.31, 95% CI = 0.12–0.75, respectively). Increased LEP levels were associated with the risk of PCOS and RPL in women with PCOS (P = 0.039, OR = 1.203, 95%CI = [1.009–1.435] and P = 0.012, OR = 1.267, 95% CI = [1.054–1.522], respectively).

**Conclusion:**

Polymorphisms rs7799039 and rs1137101 and circulating leptin and sOB-R levels were associated with infertility in Iranian women with PCOS. Further studies are needed to reveal the role of leptin in PCOS pathogenesis.

## Introduction

Polycystic ovary syndrome (PCOS) is a common endocrine and metabolic disorder, affecting 4–21% of premenopausal women [1]. It is a complex condition characterized by hyperandrogenism, oligo-ovulation or anovulation and polycystic ovarian morphologic features [1, 2]. Obesity is a common finding in PCOS patients, suggesting adipose tissue dysfunction [3]. Some evidence indicated that adipose tissue dysfunction plays a critical role in the metabolic abnormalities observed in PCOS [4]. Generally, adipose tissue, as an endocrine organ, regulates metabolic, reproductive and immune functions by secretion of various signaling molecules [5]. Dysregulated (up or down) secretion of several adipose-specific cytokines -so-called adipokines- such as adiponectin, leptin and resistin, is a common feature of dysfunctional adipose tissue in obesity and PCOS [5].

Leptin is generated mainly by adipocytes and is secreted into circulation [6]. Leptin exerts its actions in various tissues by binding to the leptin receptor [7]. There are several isoforms of leptin receptor, which are members of the cytokine family of receptors and produced by alternative splicing [8]. The soluble form of the leptin receptor (sOB-R) is comprised of only the extracellular ligand-binding domain that can bind to leptin in the circulation and modulates the level of leptin in plasma [9, 10]. Free leptin is a biologically active form in plasma and has an important role in regulating food intake, energy expenditure, lipid metabolism and insulin sensitivity [11–16]. Leptin can affect reproductive function by acting on the hypothalamic-pituitary-ovarian axis [17]. Leptin also takes part in the development of obesity, and plasma leptin concentration is found to be elevated in obese subjects [14, 18]. Obesity is a risk factor for PCOS and leptin may have major pathologic roles in obese patients with PCOS [19]. However, it has been described that leptin levels are increased in both obese and non-obese women with PCOS [20].

To date, several of the polymorphisms of the *LEP* and *LEPR* genes have been considered in the pathophysiology of metabolic syndrome [18, 21, 22]. *LEP* G2548A (rs7799039) polymorphism is located in the promoter region of the *LEP* and is associated with enhanced expression of leptin and plasma secretion from adipocytes [23]. *LEPR* Q223R point mutation (rs1137101) results in impaired leptin-binding activity [24]. Several studies have investigated the relationships between *LEP* G2548A/*LEPR* Q223R polymorphisms and obesity, diabetes, insulin resistance, dyslipidaemias and cancer [25–28]. However, few studies have addressed the association between these two polymorphisms and PCOS [29, 30]. Given the correlation between leptin, obesity, insulin action and reproductive functions, we hypothesized that plasma leptin and sOB-R levels, and also *LEP*/*LEPR* variants might be associated with PCOS as well as infertility and recurrent pregnancy loss (RPL) in PCOS patients. In this context, our study aimed to investigate the plasma levels of leptin and sOB-R, and the frequency of *LEP* rs7799039 and *LEPR* rs1137101 polymorphisms in PCOS and healthy fertile women. Moreover, in this research study, we evaluated for the first time the association of sOB-R and leptin levels, and these two single nucleotide polymorphisms with infertility and RPL in PCOS patients.

## Materials and methods

### Subjects

A total of 468 women (324 PCOS and 144 control) were included in this study. PCOS group was divided into 199 infertile cases and 125 patients with a history of RPL. The study participants were consecutively recruited from May 2017 to January 2018 at Ibn Sina Infertility Center, Tehran, Iran. All participants signed the informed consent form prior to participation in the study. This study was approved by the Ethics Committee of Ibn Sina Infertility Center (IR.ACER.Avicenna.Res.1395.6). All procedures performed in studies involving human participants were in accordance with the ethical standards of the institutional and/or national research committee and with the 1975 Helsinki declaration as revised in 2008. The diagnosis of PCOS was based on finding at least two out of three of the following criteria: menstrual disorders, hyperandrogenism and the presence of polycystic ovaries on ultrasound (Rotterdam criteria) [31]. Normal control subjects were healthy fertile women with regular menstrual cycles, no signs of hyperandrogenism and without a history of PCOS. Exclusion criteria were: diabetes mellitus, cardiovascular diseases, hypertension, current pregnancy, and metabolic disorders. Also, we excluded participants who were on hormones or other therapies that affect metabolic or endocrine functions.

### Laboratory measurements

The blood sample was obtained from subjects after a 12-h overnight fast. All samples were analyzed in term of fasting blood glucose (FBG), triglycerides (TG), total cholesterol (TC), low-density lipoprotein cholesterol (LDL-C), high-density lipoprotein cholesterol (HDL-C), fasting serum insulin, serum levels of follicle-stimulating hormone (FSH), luteinizing hormone (LH) and free testosterone (FT), as reported previously [32, 33]. Circulating levels of leptin and leptin receptor were measured using commercial ELISA kits (RD191001100, RD194002100, Biovedor, Czech Republic), the minimum detectable values were 0.2 and 0.05 ng/mL, respectively. The intra- and inter-assay coefficients of variation (CV) of leptin were 4% and 6% and for leptin receptor were 7% and 9%.

### Genetic analysis

Genomic DNA was isolated from peripheral blood leukocytes according to the standard method [34]. *LEP* rs7799039 and *LEPR* rs1137101 SNPs were genotyped using polymerase chain reaction (PCR) -restriction fragment length polymorphism (RFLP) techniques without knowledge of the case or control status of the subjects. Extracted DNA was amplified by PCR in a thermal cycler (Bio Rad, USA). The reactions were carried out using with Taq DNA polymerase (QIAGEN) and the following primers: F: 5’ AACTCAACGACACTCTCCTT-3’ and R: 5’-TGAACTGACATTAGAGGTGAC-3’ for the *LEP* gene and F: 5’-GCCTAATCC AGTATTTTCATATCTG-3’ and R: 5’-GCCACTCTTAATACC CCCAGTAC-3’ for *LEPR* gene polymorphism. The PCR amplification with 55°C annealing temperature for 30 s and 35 cycles was used for *LEP* rs7799039 and *LEPR* rs1137101. PCR products were incubated for 3h at 37°C with 5 U HhaI or MspI restriction enzymes for the *LEP* G2548A and *LEPR* Q223R polymorphisms, respectively. Then, the restriction fragments were resolved on a 2% agarose gel. In the case of *LEP* genotyping, subjects having G allele were digested and resulted in two bands of 187 bp and 267 bp, while those having A allele (without restriction site) produced a 454 bp fragment. Concerning *LEPR* genotyping, the digestion of the G allele produced two fragments with the length of 189 and 164 bp, while the resulting fragment of the A allele was 353 bp.

### Statistical analyses

Statistical analyses were performed using IBM SPSS Statistics for Windows, Version 16.0 (Armonk, NY: IBM Corp.). The Shapiro-Wilk test was used to assess the normality of the data. The differences in continuous data between study groups were analyzed using the Independent *t*-test and ANOVA tests or their equal non-parametric tests in the case of skewed data. The Chi-Square test was used to compare the groups in case of categorical data. The correlation between leptin and sOB-R was analyzed by the Pearson correlation test. A binary logistic regression model was used to investigate the simultaneous effect of the variables on the PCOS. Multinomial logistic regression analysis was performed to assess the effects of variables between the PCOS, PCOS-infertile and PCOS-RPL groups. The significance level was considered as p<0.05.

## Results

**Fig 1:**
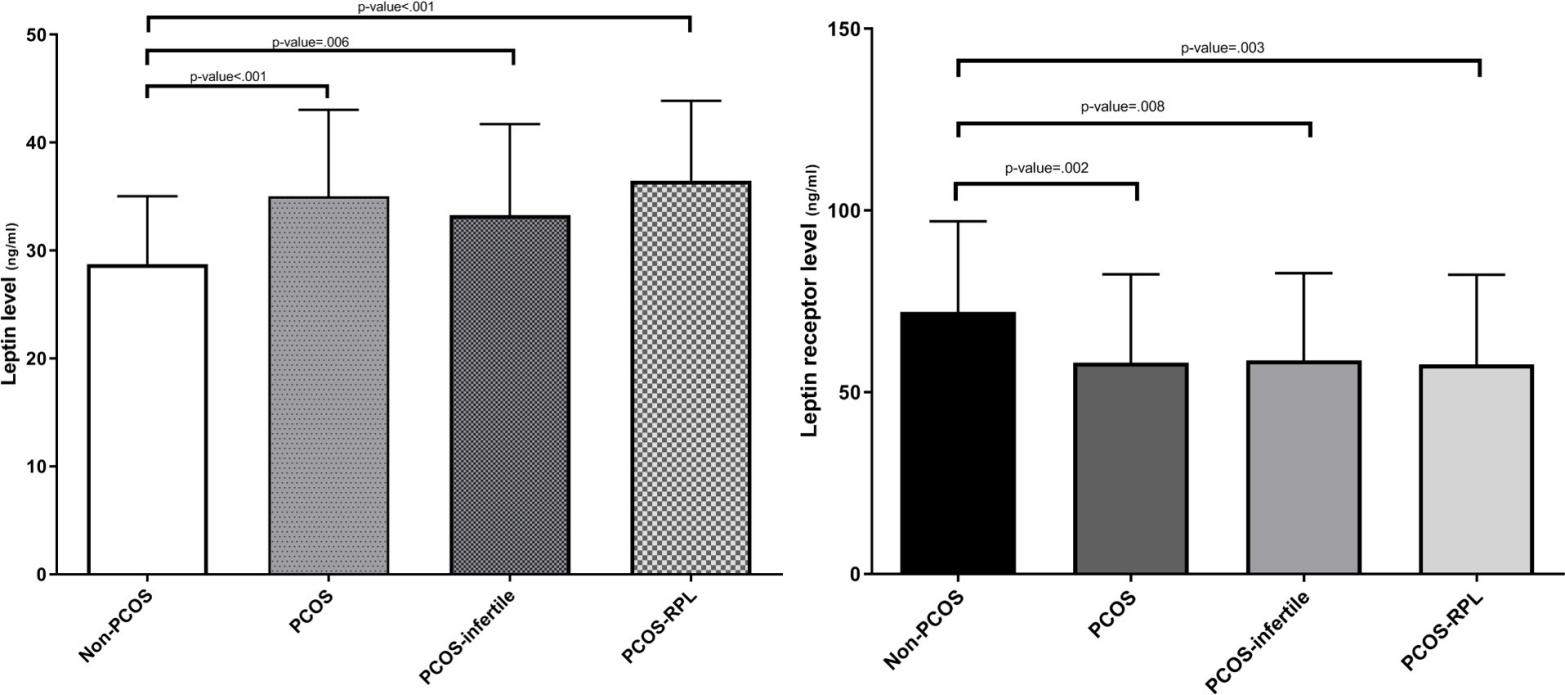
Leptin and leptin receptor levels of non-PCOS subjects and PCOS patients (PCOS-infertile and PCOS-RPL sub-groups).

**Fig 2:**
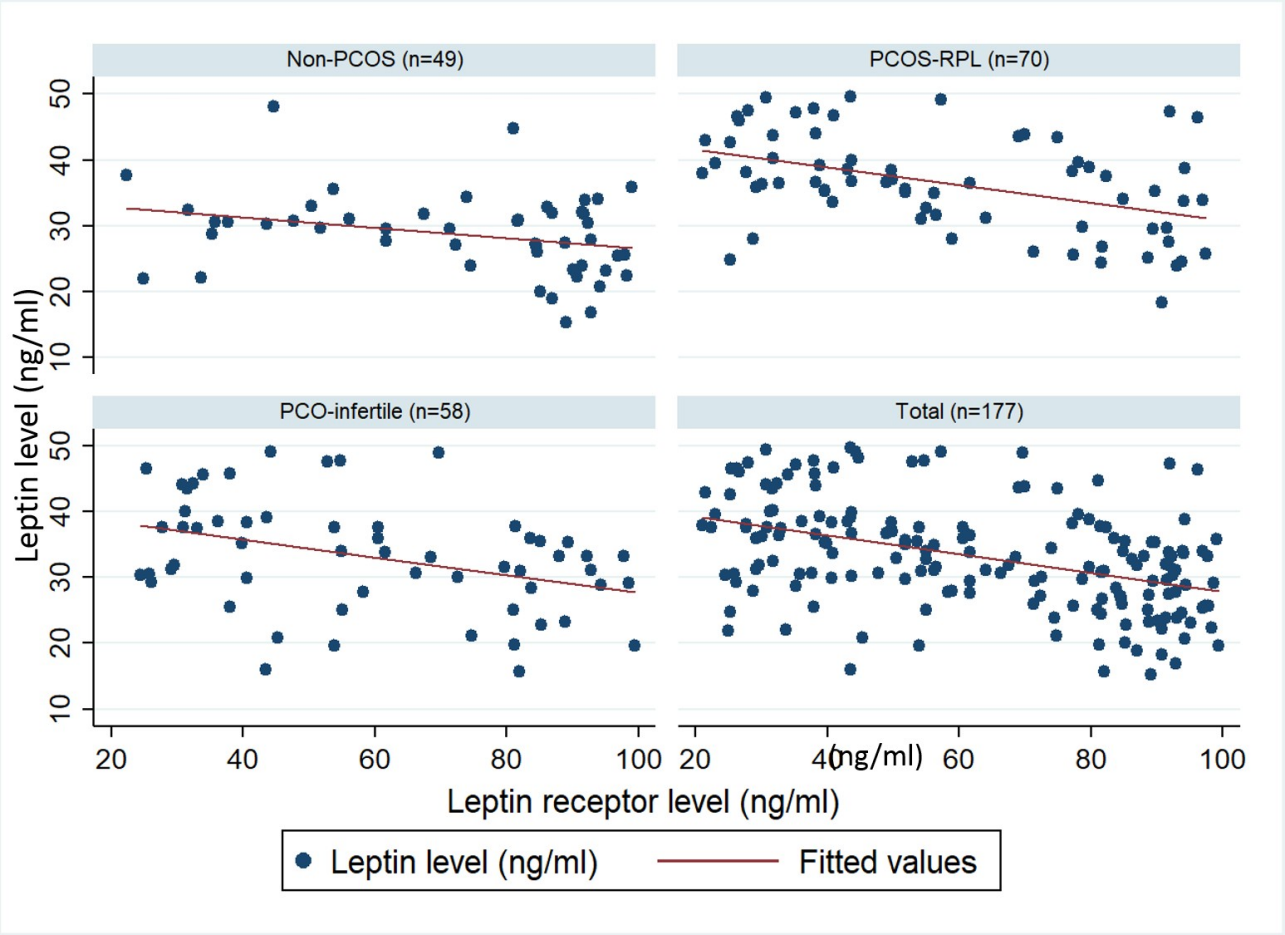
Comparison of correlation between leptin and leptin receptor levels in non-PCOS subjects and PCOS-infertile or PCOS-RPL groups, as well as all population.

The clinical characteristics and biochemical parameters of the study population are shown in Table 1 as reported in previous studies [35, 36]. Statistically significant difference was observed between the PCOS, PCOS-infertile, PCOS-RPL women and non-PCOS groups based on age, BMI, insulin and Free T. The levels of TG and LH in the PCOS patients were significantly higher than in the non-PCOS women. In addition, TG showed a higher value in PCOS-RPL compared to non-PCOS women. LH in PCOS-infertile women was significantly higher than the non-PCOS group. While, in the PCOS-RPL group, TG, and LH showed significant differences compared with PCOS-infertile women. The total number of samples which assessed for leptin and leptin receptor levels was 177 (non-PCOS = 49, PCOS-RPL = 70 and PCOS-infertile = 58). As presented in Fig 1, the PCOS women had significantly higher plasma leptin levels than the non-PCOS group (PCOS vs non-PCOS: 35.02±8.02 vs 28.73±6.29 ng/ml, P<0.001). Moreover, leptin level was significantly higher in PCOS-infertile (33.27±8.45 ng/ml) and PCOS-RPL (36.47±7.41 ng/ml) sub-groups compared to the non-PCOS group. There was a significantly lower level of sOB-R in PCOS (58.13±24.3 ng/ml), PCOS-infertile (58.74±24.04 ng/ml) and PCOS-RPL (57.62±24.67 ng/ml) compared to the non-PCOS group (72.95±22.95 ng/ml). A significant reverse correlation existed between plasma levels of leptin and sOB-R in non-PCOS (r = -0.289, P = 0.044) subjects and PCOS females with infertility (r = -0.382, P = 0.003) or RPL (r = -0.447, P<0.001) (Fig 2).

**Table 1:** Comparison of clinical characteristics between non-PCOS and PCOS subjects.

	Non-PCOS (n = 144)	PCOS (n = 324)	p-value†	PCOS-infertile (n = 199)	PCOS-RPL (n = 125)	p-value‡
**Age** (years)	31.86±5.12^ab^	29.90±4.56	<0.001	29.79±4.70^a^	30.09±4.35^b^	<0.001
**BMI** (kg/m2)	25.42±3.96^ab^	26.70±4.49	0.004	26.66±4.20^a^	26.75±4.96^b^	0.017
**FBS** (mg/dL)	90.30±9.66	89.67±9.51	0.517	89.80±9.67	89.45±9.27	0.771
**Insulin** (μU/mL)	3.26±1.90^ab^	5.62±3.83	<0.001	5.62±3.68^a^	5.62±4.05^b^	< .001
**TG** (mg/dL)	117.41±39.80^a^	127.26±57.21	0.035	120.60±57.16^b^	137.79±55.90^ab^	0.003
**TC** (mg/dL)	164.92±39.74	172.15±35.61	0.055	170.03±33.61	175.51±38.49	0.069
**LDL-C** (mg/dL)	99.20±30.86	98.28±29.15	0.760	97.33±27.25	99.80±32.02	0.738
**HDL-C** (mg/dL)	46.66±7.22	45.04±11.35	0.068	45.10±11.88	44.95±10.50	0.301
**Free-T** (pg/mL)	1.53±.33^ab^	3.24±1.13	<0.001	3.15±.92^a^	3.32±1.31^b^	<0.001
**LH** (IU/L)	6.68±2.62^a^	7.56±4.83	0.025	8.62±5.50^ab^	5.85±2.77^b^	<0.001
**FSH** (IU/L)	8.44±2.35	6.56±3.54	<0.001	6.11±2.17	7.30±7.93	<0.001

**†**Independent t test for comparison between PCOS and Non-PCOS groups

‡One-way ANOVA with Bonferroni post hoc test for PCOS-infertile and PCOS-RPL subgroups compared to Non-PCOS.

Significant differences among pairwise groups with Bonferroni’s approach indicated by similar uppercase letters (such as a and b).

PCOS, Poly Cystic Ovary Syndrome; RPL, recurrent pregnancy loss; BMI, Body Mass Index; FBS, Fasting Blood Sugar; TG, Triglyceride; TC, Total Cholesterol; LDL-C, Low Density Lipoprotein Cholesterol; HDL-C, High Density Lipoprotein Cholesterol; T, Testosterone; LH: luteinizing hormone; FSH: follicle-stimulating hormone.

The results of genotype and allele frequencies of *LEP* (rs7799039) and *LEPR* (rs1137101) polymorphisms are given in Table 2. *LEP* polymorphism (rs7799039) was genotyped for 80 and 268 samples in non-PCOS and PCOS groups, respectively. Also, *LEPR* polymorphism (rs1137101) was detected for 83 and 263 samples in non-PCOS and PCOS groups, respectively. Allelic and genotypic frequencies of rs7799039 and rs1137101 polymorphisms were in the Hardy-Weinberg equilibrium (P>0.05). The analysis of rs7799039 and rs1137101 gene polymorphisms revealed significant differences in GG genotype in PCOS-infertile women as compared to non-PCOS subjects (P = 0.043, OR = 0.47, 95% CI = 0.22–0.97, and P = 0.01, OR = 0.31, 95% CI = 0.12–0.75, respectively). Our data also showed that there were significant differences in allelic (G) and genotypic (GG) frequencies for the *LEPR* (rs1137101) polymorphism in PCOS women when compared with the non-PCOS subjects (P = 0.033, OR = 0.67, 95% CI = 0.46–0.96 and P = 0.02, OR = 0.39, 95% CI = 0.18–0.86, respectively). Besides, a significant difference in frequency of G allele in PCOS-infertile vs. non-PCOS group was shown (P = 0.025, OR = 0.64, 95% CI = 0.43–0.94).

**Table 2:** Genotypic and allelic distribution of *LEP* (rs7799039) and *LEPR* (rs1137101) polymorphisms in PCOS and non-PCOS groups.

		Non-PCOS	PCOS	PCOS-infertile	PCOS-RPL	PCOS vs Non-PCOS^a^		PCOS-infertile vs Non-PCOS^b^		PCOS-RPL vs Non-PCOS^b^	
		N (%)	N (%)	N (%)	N (%)	OR(CI)	p-value	OR(CI)	p-value	OR(CI)	p-value
**Leptin genotype (rs7799039)**	**AA**	25 (20.7)	96 (79.3)	60 (49.6)	36 (29.8)	ref		ref		ref	
	**GA**	32 (20.4)	125 (79.6)	82 (52.2)	43 (27.4)	1.02 (0.56–1.82)	0.954	1.06 (0.57–1.98)	0.836	0.93 (0.47–1.85)	0.843
	**GG**	23 (32.9)	47 (67.1)	26 (37.1)	21 (30.0)	0.53 (0.27–1.03)	0.063	0.47 (0.22–0.97)	**0.043**	0.63 (0.29–1.38)	0.253
**Leptin receptor genotype (rs1137101)**	**AA**	32 (20.3)	126 (79.7)	81 (51.3)	45 (28.5)	ref		ref		ref	
	**AG**	37 (24.3)	115 (75.7)	76 (50.0)	39 (25.7)	0.78 (0.46–1.35)	0.387	0.81 (0.46–1.43)	0.470	0.75 (0.39–1.41)	0.376
	**GG**	14 (38.9)	22 (61.1)	11 (30.6)	11 (30.6)	0.39 (0.18–0.86)	**0.02**	0.31 (0.12–0.75)	**0.01**	0.55 (0.22–1.38)	0.210
**Leptin allele (rs7799039)**	**A**	82 (20.6)	317 (79.4)	202 (50.6)	115 (28.8)	ref		ref		ref	
	**G**	78 (26.3)	219 (73.7)	134 (45.1)	85 (28.6)	0.72 (0.50–1.03)	0.077	0.69 (0.47–1.01)	0.062	0.77 (0.51–1.18)	0.777
**Leptin receptor allele (rs1137101)**	**A**	101 (21.6)	367 (78.4)	238 (50.9)	190 (27.6)	ref		ref		ref	
	**G**	65 (29.0)	159 (71.0)	98 (43.8)	61 (27.2)	0.67 (0.46–0.96)	**0.033**	0.64 (0.43–0.94)	**0.025**	0.73 (0.47–1.13)	0.166

a: Differences were examined by Binary logistic model and reference category was non-PCOS.

b: Differences were examined by Multinomial logistic model; reference category is non-PCOS.

Number of genotyped samples for *LEP* polymorphism (rs7799039): non-PCOS = 80 and PCOS groups = 268.

Number of genotyped samples for *LEPR* polymorphism (rs1137101): non-PCOS = 83 and PCOS groups = 263.

OR, Odds Ratio; CI, Confidence Interval; ref, reference; N, number.

To find some details about the role of *LEP* and *LEPR* gene polymorphisms in the pathological processes of PCOS, we evaluated their associations with clinical characteristics. According to the post-hoc of Bonferroni, there were no significant associations between clinical characteristics and the genotypes of *LEP* (rs7799039) and *LEPR* (rs1137101) polymorphisms in all studied population (S1 Table). In addition, no significant associations between the genotypes of rs7799039 and rs1137101 polymorphisms and clinical characteristics were observed in PCOS-infertile and PCOS-RPL subgroups (data not shown).

Multi-nominal logistic regression analysis was applied to determine the odds ratios and 95% confidence intervals (CIs) of women with PCOS-infertile and PCOS-RPL, in reference to the non-PCOS group (Table 3). Binary logistic regression was also used to explore the effect of variables on PCOS. Plasma level of free testosterone was associated with increased risk of diagnosing PCOS females (44.803, 95% CI [4.909–408.888]) with infertility (35.759, 95% CI [3.879–329.606]) or RPL (43.785, 95% CI [4.751–403.504]). Importantly, it is demonstrated that leptin elevated the risk of PCOS (1.203, 95%CI [1.009–1.435]) as well as RPL related PCOS (1.267, 95% CI [1.054–1.522]) in females.

**Table 3:** *LEP* (rs7799039) and *LEPR* (rs1137101) polymorphisms and the risk of PCOS, PCOS-infertile, and PCOS-RPL.

Variables	PCOS^a^		PCOS-infertile^b^		PCOS-RPL^b^	
	Adjusted OR (95% CI)	P	Adjusted OR (95% CI)	P	Adjusted OR (95% CI)	P
**Age** (years)	0.796 (0.574–1.105)	0.172	0.825(0.589–1.155)	0.262	0.79 (0.565–1.104)	0.167
**BMI** (kg/m2)	0.723 (0.462–1.131)	0.155	0.711 (0.448–1.127)	0.146	0.694 (0.438–1.1)	0.120
**Insulin** (μU/mL)	1.492 (0.897–2.481)	0.123	1.536 (0.913–2.584)	0.106	1.522 (0.905–2.561)	0.114
**TG** (mg/dL)	0.998 (0.97–1.027)	0.9	0.993 (0.964–1.022)	0.622	1.002 (0.974–1.031)	0.872
**Free-T** (pg/mL)	44.803 (4.909–408.888)	**0.001**	35.759 (3.879–329.606)	0.**002**	43.785 (4.751–403.504)	0.**001**
**LH** (IU/L)	1.093 (0.812–1.47)	0.557	1.21 (0.891–1.643)	0.222	1 (0.729–1.37)	0.999
**FSH** (IU/L)	0.989 (0.715–1.366)	0.945	0.88 (0.624–1.242)	0.468	1.039 (0.748–1.443)	0.821
**Leptin_genotype AA (ref)**			.	.	.	.
**Leptin_genotype(GG)**	0.164 (0.006–4.216)	0.275	0.176 (0.006–4.826)	0.304	0.176 (0.006–5.051)	0.311
**Leptin_genotype (GA)**	2.057 (0.2–21.105)	0.544	3.452 (0.314–37.971)	0.311	1.469 (0.132–16.356)	0.755
**Leptin_receptor_genotype AA (ref)**			.	.	.	.
**Leptin_receptor_genotype(GG)**	0.215 (0.01–4.676)	0.328	0.152 (0.006–3.934)	0.256	0.261 (0.011–5.927)	0.399
**Leptin_receptor_genotype (AG)**	0.592 (0.055–6.392)	0.666	0.926 (0.082–10.517)	0.951	0.517 (0.046–5.843)	0.594
**Leptin_level** (ng/ml)	1.203 (1.009–1.435)	**0.039**	1.127 (0.94–1.352)	0.196	1.267 (1.054–1.522)	**0.012**
**Leptin_receptor_level** (ng/ml)	0.999 (0.943–1.059)	0.977	0.988 (0.932–1.047	0.682	1.002 (0.946–1.063)	0.937

Age and BMI as covariates were adjusted.

a: Binary logistic results, reference category is non-PCOS.

b: Multinomial logistic results, reference category is non-PCOS.

## Discussion

Adipose tissue dysfunction is possibly involved in the development of PCOS [4]. It may negatively affect metabolic health through dysregulated secretion of some adipokines including leptin [5]. There are two forms of leptin in the circulation, an sOB-R-bound form and a free (or active) one [9]. A growing body of evidence demonstrated that leptin changes are associated with metabolic disorders [14]. The present study showed a significantly higher level of free leptin in the PCOS group and also infertile and RPL subgroups as compared to the healthy subjects. Significant differences in some clinical characteristics between the study groups may be justified by metabolic effects of leptin. It has been indicated that elevated serum leptin levels are associated with fasting insulin, BMI and TG [37–39]. In addition, the modulatory effects of leptin on hypothalamic-pituitary-gonadal axis functions have been suggested [40]. The level of sOB-R was significantly lower in PCOS, PCOS-infertile and PCOS-RPL groups than in the control group. In conformity with the findings of the current study, an increasing number of studies have reported an increased leptin level and decreased sOB-R level in PCOS women [41–46], however, some studies showed no significant difference in leptin and sOB-R between PCOS women and healthy subjects [47–50]. This discrepancy could be likely due to the race, complex and heterogeneous nature of PCOS and sample size. Furthermore, a study on 40 women with PCOS and 36 healthy women in the Iranian population reported enhanced leptin concentrations in PCOS, which was similar to the findings of the current study [39]. In agreement with the results of the present study, an inverse association between leptin and sOB-R was observed in some studies [41, 46]. It has been shown that the level of the free form of leptin in obesity increases due to small sOB-R concentrations [51]. Indeed, sOB-R may be reduced to compensate for the observed defective leptin action and leptin resistance in PCOS women [41]. So, it can be assumed that hyperleptinemia may be a result of decreased sOB-R levels in PCOS women. Anyway, hyperleptinemia could influence the reproductive functions and may lead to infertility by affecting the secretion of GnRH, FSH and LH from the hypothalamus/hypophysis, oocyte maturation and steroidogenesis [52]. In addition, some studies suggest that leptin has a crucial role in embryo–maternal cross-talk and implantation processes through the development of the placenta and endometrial receptivity [53, 54]. However, the exact role of leptin and sOB-R in the development of infertility and RPL in women with PCOS is not fully clear. In line with our data, the results of a study by Jahromi et.al showed that infertile women with PCOS had an increased level of leptin as compared to the control group [55]. Until now, no study has examined the association of leptin and sOB-R with PCOS-RPL. The present study demonstrates for the first time the positive and negative correlation of leptin and sOB-R levels with PCOS-RPL, respectively. Recently, Mutalib et.al reported a significantly increased level of leptin in pregnant women with a history of RPL [56]. Although Plowden et.al reported the potential role of leptin in adverse pregnancy outcomes [57], the exact effect of leptin level on RPL -in PCOS and/or non-PCOS women- remains to be elucidated.

The results of logistic regression indicated that leptin elevates the risk of PCOS and also RPL in PCOS women. This analysis also showed that the level of free testosterone is associated with an increased risk of PCOS, PCOS-related infertility or RPL. In agreement with these results, Rizk et.al reported that there was a significant association between free testosterone and leptin with PCOS disease [46].

The rs7799039 polymorphism of the *LEP* gene is located in the promoter region and influences the serum leptin concentrations [23]. Although some studies reported a good correlation between *LEP* gene genotypes and serum leptin levels [23, 58], we did not find a correlation between *LEP* polymorphisms and the serum concentrations of leptin. This discrepancy may be due to the difference in the studied population. Although the association of rs7799039 and rs1137101 polymorphisms with some metabolic disorders including obesity, insulin resistance and dyslipidemia has been evaluated [25–28], few studies have been carried out on the association between these polymorphisms and PCOS [29, 30]. The present study indicates for the first time the association of rs7799039 and rs1137101 polymorphisms with PCOS-related infertility in Iranian women. In the current study, a significant difference was observed in the GG genotype frequency of rs7799039 polymorphism between normal subjects and infertile PCOS women. The allele frequency of rs7799039 polymorphism was not significantly different between PCOS cases and non-PCOS women. A similar result was reported in Bahraini and Tunisian women [30]. In the case of rs1137101 polymorphism of the *LEPR* gene, allelic (G) and genotypic (GG) frequencies were significantly different in PCOS cases and non-PCOS subjects. The results of this study showed that rs1137101 polymorphism of the *LEPR* gene is associated with PCOS and PCOS-related infertility. There are conflicting data about the association between this polymorphism and PCOS in the different populations [29, 59]. Li et.al showed that the G allele of rs1137101polymorphism is significantly associated with the decreased risk of PCOS in the Korean population [60]. Recently, a study by Dallel et.al reported significantly different allele frequencies of rs1137101 polymorphism between PCOS subjects and control women from Bahrain [29]. They also indicated that the G allele of rs1137101 polymorphism is associated with the risk of PCOS in Bahraini women, while this association was not found in Tunisians [29]. Moreover, Daghestani et.al showed no significant association between rs1137101 polymorphism of *LEPR* gene and PCOS in Saudi women [59]. According to the results of this study and other research, it can be assumed that the association of *LEP* and *LEPR* variants with PCOS–and also PCOS-related infertility and RPL- is dependent on the ethnic/racial background of the study population.

In this study, we investigated for the first time the level of leptin and sOB-R and the frequency of rs7799039 and rs1137101 SNPs in PCOS-infertile and PCOS-RPL women. However, there were a number of limitations, such as the relatively small sample size, and lack of access to different phenotypes of PCOS. Although RFLP method is relatively accurate and reliable for the detection of DNA polymorphisms, the precision of that can be verified by another method such as DNA sequencing. By considering diverse phenotypes of PCOS in future studies in different populations with larger sample sizes, the detailed mechanism of leptin action in the pathogenesis of PCOS could be found.

## Conclusion

In conclusion, this study showed a significantly higher level of leptin and lower level of sOB-R in the PCOS group as well as PCOS-infertile and PCOS-RPL subgroups in comparison with non-PCOS women. A significant reverse correlation was revealed between plasma levels of leptin and sOB-R in non-PCOS and PCOS women. In addition, it was found that leptin levels increase the risk of PCOS and RPL related PCOS. So, it can be suggested that hyperleptinemia observed in PCOS women may be due to reduced sOB-R levels, which in turn may lead to infertility and RPL in PCOS patients. Moreover, the present study demonstrated a significant association between the GG genotype of rs7799039 and rs1137101 polymorphisms and PCOS-related infertility. However, further investigations are necessary to reveal the potential implications of the *LEP* and *LEPR* gene polymorphisms in the pathological processes of PCOS.

## Supporting information

S1 Table Association of the clinical characteristics and level of leptin and leptin receptor with genotypic frequencies for leptin (rs7799039) and leptin receptor (rs1137101) polymorphisms in all population. (DOCX)

## Abbreviations

BMI: Body Mass Index
CI: Confidence Interval
FBG: Fasting Blood Glucose
FSH: Follicle-Stimulating Hormone
FSHR: Follicle-Stimulating Hormone Receptor
FT: Free Testosterone
GnRHR: Gonadotropin-Releasing Hormone Receptor
HDL-C: High-Density Lipoprotein Cholesterol
HOMA-IR: Homeostasis Model Assessment of IR
LDL-C: Low-Density Lipoprotein Cholesterol
LH: Luteinizing Hormone
OR: Odds Ratio
PCOS: Polycystic Ovary Syndrome
PCR: Polymerase Chain Reaction
RFLP: Restriction Fragment Length Polymorphism
RPL: recurrent pregnancy loss
SD: Standard Deviation
TC: Total Cholesterol
sOB-R: Soluble Form of the Leptin Receptor
TG: Triglyceride
