# Supplementary material for: Association of leptin G2548A and leptin receptor Q223R polymorphisms and their serum levels with infertility and recurrent pregnancy loss in Iranian women with polycystic ovary syndrome
Source: PLoS One. 2021 Aug 18;16(8):e0255920. doi: 10.1371/journal.pone.0255920 (PMC8372961; doi:10.1371/journal.pone.0255920)
Supplement: S1 Table — (DOCX) [file pone.0255920.s001.docx]

| **Clinical characteristics** | *LEP* **(rs7799039)** | | | | *LEPR* **(rs1137101)** | | | |
| --- | --- | --- | --- | --- | --- | --- | --- | --- |
|  | **AA** | **GA** | **GG** |  | **AA** | **AG** | **GG** |  |
|  | **Mean±SD** | **Mean±SD** | **Mean±SD** | **p-value** | **Mean±SD** | **Mean±SD** | **Mean±SD** | **p-value** |
| **Age** (years) | 30.69±4.97 | 31.01±4.62 | 31.29±4.98 | 0.698 | 30.76±4.97 | 31.27±4.7 | 30.6±4.21 | 0.578 |
| **BMI** (kg/m2) | 26.1±3.91 | 26.64±4.99 | 27.04±5.08 | 0.396 | 26.46±4.85 | 26.95±4.76 | 25.23±3.44 | 0.144 |
| **FBS** (mg/dL) | 89.48±9.8 | 89.63±9.85 | 88.45±8.03 | 0.685 | 89.63±9.43 | 89.27±9.85 | 89.28±8.59 | 0.943 |
| **Insulin** (μU/mL) | 5.28±4.21 | 5.16±3.68 | 4.98±3.05 | 0.874 | 4.95±3.48 | 5.48±4.24 | 4.29±2.36 | 0.196 |
| **TG** (mg/dL) | 119.47±51.65 | 124.89±55.31 | 130.39±57.25 | 0.411 | 126.19±60.69 | 122.46±48.42 | 113.83±42.79 | 0.459 |
| **TC** (mg/dL) | 170.99±37.36 | 169.23±38.02 | 170.46±33.24 | 0.922 | 169.4±36.78 | 171.51±35.28 | 164.03±38.24 | 0.539 |
| **LDL-C**(mg/dL) | 96.97±30.21 | 98.24±28.69 | 96.89±28.51 | 0.921 | 97.4±31.35 | 98.86±27.48 | 90.42±27.05 | 0.317 |
| **HDL-C** (mg/dL) | 45.38±13.42 | 45.42±9.68 | 45.19±9.19 | 0.989 | 46.04±12.62 | 44.64±9.47 | 45.88±9.32 | 0.527 |
| **Free-T** (pg/mL) | 2.8±1.28 | 2.86±1.25 | 2.91±1.27 | 0.916 | 2.96±1.22 | 2.84±1.28 | 2.3±1.29 | 0.106 |
| **LH** (IU/L) | 7.58±5.16 | 7.06±4.57 | 7.56±4.58 | 0.653 | 7.13±3.99 | 7.85±5.77 | 6.3±3.14 | 0.198 |
| **FSH** (IU/L) | 6.89±3.31 | 6.6±3.26 | 6.98±3.66 | 0.707 | 6.54±3.43 | 6.96±3.29 | 7.48±3.14 | 0.292 |
| **Leptin level** (ng/ml) | 33.18±8.04 | 33.61±8.61 | 32.71±7.72 | 0.862 | 33.55±7.97 | 33.87±8.4 | 30.52±8.12 | 0.260 |
| **Leptin receptor level** (ng/ml) | 62.77±24.67 | 61.48±24.17 | 61.56±27.06 | 0.953 | 61.08±25.07 | 60.41±24.8 | 68.85±24.08 | 0.389 |

Differences between cases and controls are obtained based on the ANOVA.

Significant associations among pairwise categories indicated by similar uppercase symbol * and #.
